# SRY-negative 46,XX testicular disorder of sex development (de la Chapelle syndrome) presenting with primary infertility: a case report

**DOI:** 10.5935/1518-0557.20260040

**Published:** 2026

**Authors:** Shubhi Srivastava, Sangita Sharma

**Affiliations:** 1 Department of Reproductive Medicine and Surgery, Mahatma Gandhi Medical College and Hospital, Jaipur, Rajasthan, India; 2 Department of Reproductive Medicine and Surgery, Centre Head, Jaipur Fertility and Research Centre, Mahatma Gandhi Medical College and Hospital, Jaipur, Rajasthan, India

**Keywords:** azoospermia, 46,XX karyotype, SRY-negative, de la Chapelle syndrome, male infertility, hypergonadotropic hypogonadism

## Abstract

De la Chapelle syndrome describes phenotypic males with a 46,XX karyotype, most commonly caused by translocation of the SRY gene. The SRY-negative variant is exceptionally rare and invariably associated with non-obstructive azoospermia. Awareness of this condition is important for infertility specialists, as genetic evaluation can establish an accurate diagnosis early in the workup and guide appropriate counselling. We report the case of a 30-year-old man who presented with primary infertility. Clinical examination revealed small testes with an otherwise normal male phenotype. Semen analysis confirmed azoospermia, and hormonal testing showed elevated gonadotropins with borderline testosterone levels. Imaging demonstrated reduced testicular volume without malignant features. Karyotype analysis revealed a 46,XX constitution, and molecular testing confirmed the absence of the SRY gene, establishing the diagnosis of SRY-negative de la Chapelle syndrome. Based on these findings, the patient was counselled regarding the futility of autologous sperm retrieval and was advised on donor-assisted reproductive options. Written informed consent was obtained for publication. This case highlights the value of incorporating genetic testing into the evaluation of non-obstructive azoospermia. Early recognition avoids unnecessary invasive interventions such as testicular sperm extraction and allows couples to receive timely counselling about realistic reproductive options. Although natural fertility cannot be achieved, assisted reproduction with donor sperm offers a viable pathway to parenthood. Comprehensive care, including endocrine monitoring, psychosocial support, and multidisciplinary management, ensures optimal outcomes for affected individuals.

## INTRODUCTION

Infertility affects ~15% of couples worldwide; male factors account for approximately 50% of cases (Bereketoğlu *et al.*, 2022; Tüttelmann *et al.*, 2018). Non-obstructive azoospermia represents one of the most severe forms of male infertility, with chromosomal abnormalities detected in approximately 10-15% of affected men and Y-chromosome microdeletions in 5-13% depending on the cohort and diagnostic methods (Bereketoğlu *et al.*, 2022; Tüttelmann *et al.*, 2018).

De la Chapelle syndrome (46,XX testicular DSD) is a rare genetic cause of non-obstructive azoospermia, with an estimated incidence of 1 per 20,000-25,000 male births (Chen *et al.*, 2019). It is classified into SRY-positive and SRY-negative variants. The SRY-negative form accounts for 10-20% of cases and may result from alternative genetic mechanisms, including duplications or regulatory changes in SOX9, gain-of-function alterations in SOX3, NR5A1 variants, WT1 mutations, and other copy-number changes affecting testis-determining genes (Li *et al.*, 2014; Croft *et al.*, 2018; Mengen *et al.*, 2020).

Beyond infertility, these patients may present with hypogonadism requiring hormone replacement therapy (HRT) to preserve secondary sexual characteristics, bone health, libido, and metabolic balance (Délot & Vilain, 2022). In SRY-negative 46,XX individuals, the absence of Y-chromosomal sequences, including the gonadoblastoma locus on the Y chromosome (GBY) region, means the classic gonadoblastoma-associated malignancy risk is not a major concern; however, careful long-term follow-up is still recommended, as the true neoplastic risk in this subset is not fully defined. We report an SRY-negative 46,XX testicular DSD case presenting with primary infertility, and we review diagnostic considerations and management implications.

## CASE DESCRIPTION (Table 1)

A 30-year-old phenotypic male presented to Jaipur Fertility and Research Centre (part of Mahatma Gandhi Medical College and Hospital, Jaipur) with a five-year history of primary infertility. The patient’s 30-year-old wife underwent a complete fertility workup, which revealed regular menstrual cycles, normal hormonal profiles, a total antral follicle count of 16-20 and no uterine or ovarian abnormalities on transvaginal ultrasound.

**Table 1:** Laboratory, Imaging, and Genetic Investigations

Date (2024)	Investigation	Result	Reference Range/Remarks
10 May	Semen analysis 1	Azoospermia (no spermatozoa seen even after centrifugation 3000 x g for 15 min)	WHO 6^th^ edition (2021)
05 July	Semen analysis 2 (repeat)	Azoospermia - confirmed	WHO 6^th^ edition (2021)
08 June	Endocrine profile - FSH - LH - Testosterone - Prolactin - TSH	74.3 mIU/mL 38.4 mIU/mL 344.8 ng/dL (11.9 nmol/L) 7.6 ng/mL 2.14 mIU/L	1.5-12 mIU/mL 1.7-8.6 mIU/mL 300-1000 ng/dL (10.4-34.7 nmol/L) 4-15 ng/mL 0.4-4.0 mIU/mL
02 June	Scrotal ultrasonography	Bilaterally small testes (right 3.2 mL, left 2.8 mL) with microlithiasis; epididymides normal; no varicocele	High-resolution scrotal USG
12 July	Karyotype (G-banded 550-resolution)	46,XX (normal female karyotype, no structural abnormality)	NABL-accredited lab
18 July	FISH (SRY probe)	Absence of SRY signal; no translocation to X or autosomes detected	SRY (Yp11.31, orange); DYZ1 (Yq12, green); DXZ1 (Xp11.1-q11.1, blue)

The patient was a phenotypic male with normal virilization (facial hair, deep voice) and normal muscle mass; there was no history of pubertal delay or gynecomastia. On physical examination, his height and weight were within normal limits; his stretched penile length measured 10 cm. Genital examination revealed bilaterally small testes (3-4 mL by Prader orchidometer), with both vasa deferentia palpable and no varicocele or hydrocele.

Scrotal ultrasound using a high-frequency linear transducer confirmed small testicular volumes (right testis: 3.2 mL; left testis: 2.8 mL) with bilateral scattered intratesticular microlithiasis and reduced intratesticular vascularity. Two semen analyses (May 2024 and July 2024) were performed in accordance with the WHO Laboratory Manual for the Examination and Processing of Human Semen, 6th edition (2021). Each sample underwent centrifugation at 3000 x g for 15 minutes, followed by post-centrifugation examination, which confirmed azoospermia. Transrectal ultrasonography (TRUS) was not performed, as clinical examination and scrotal ultrasonography findings were adequate for evaluation.

Endocrine evaluation revealed FSH 74.3 mIU/mL (reference 1.5-12), LH 38.4 mIU/mL (1.7-8.6), serum total testosterone 344.8 ng/dL (300-1000 ng/dL / 10.4-34.7 nmol/L), prolactin 7.6 ng/mL (4-15), and TSH 2.14 mIU/L (0.4-4.0 mIU/L). These results indicated hypergonadotropic hypogonadism. Cytogenetic analysis of peripheral-blood lymphocytes (G-banded metaphases at 550-band resolution; NABL-accredited laboratory, Jaipur) demonstrated a 46,XX karyotype with no structural chromosomal abnormalities. Fluorescence in situ hybridization (FISH) performed at GSS Diagnostics, Jaipur, using SRY (Yp11.31, orange), DYZ1 (Yq12, green), and DXZ1 (Xp11.1-q11.1, blue) probes on 100 interphase and 10 metaphase cells revealed complete absence of SRY signal with no evidence of translocation to the X chromosome or autosomes. Y-chromosome microdeletion (AZF region) and array-CGH or targeted gene sequencing were not performed due to financial constraints; the patient was counselled accordingly. After multidisciplinary counselling regarding reproductive and endocrine options, the couple elected donor sperm insemination (IUI). Psychological counselling was provided by the clinical psychologist attached to the Department of Reproductive Medicine and Surgery, Mahatma Gandhi Medical College & Hospital, Jaipur, focusing on infertility-related stress, and partner communication. Testosterone replacement therapy (TRT) was deferred because testosterone was at the low-normal level and the patient had adequate virilization and libido. He was advised annual endocrine and bone-health monitoring.

## DISCUSSION

De la Chapelle syndrome is characterized by a male phenotype in individuals with a 46,XX karyotype. In SRY-negative cases, testicular differentiation occurs through alternative genetic mechanisms such as SOX9 enhancer duplications or regulatory region copy-number variations, SOX3 gene duplications, and pathogenic variants in NR5A1 or WT1 that up-regulate the testicular pathway (Li *et al.*, 2014; Croft *et al.*, 2018; Mengen *et al.*, 2020). These changes drive Sertoli-cell differentiation despite the absence of SRY. The gonadal phenotype is variable, ranging from normal-appearing testes to dysgenetic gonads, but spermatogenesis is invariably absent because germ-cell development requires genes located on the Y-chromosome AZF region. Despite elevated gonadotropins, some patients maintain testosterone within or near the normal range, likely due to residual Leydig cell activity (Rajender *et al.*, 2006).

Endocrine management focuses on maintaining eugonadal testosterone levels to preserve libido, bone mineral density, and metabolic health. Long-term follow-up should include annual serum testosterone review, bone-density evaluation, and counselling on metabolic risk factors. Testosterone replacement is indicated when levels fall below physiologic range or when clinical symptoms of hypogonadism appear.

Tumor risk in SRY-negative cases is lower than in Y-positive DSD because the GBY (gonadoblastoma locus on Y) region is absent; however, data on lifetime risk are sparse (Wisniewski *et al.*, 2019). A practical recommendation is patient education for monthly testicular self-examination and periodic ultrasonography-initially every 12 months and subsequently at individualized intervals-especially when dysgenetic features or microlithiasis are present. In the present case, bilateral testicular microlithiasis was noted, a finding occasionally associated with impaired spermatogenesis but not proven premalignant. Annual ultrasonographic surveillance and self-examination were advised as part of long-term care.

Management extends beyond reproductive counselling. Hypogonadism requires timely hormone-replacement therapy to maintain sexual function, bone density, and metabolic stability (Délot & Vilain, 2022). Fertility restoration with autologous sperm is very unlikely; therefore, donor sperm insemination, assisted reproduction with donor gametes, or adoption remain the main reproductive alternatives (Raguraman *et al.*, 2024). Genetic counselling is vital to explain the diagnosis, inheritance patterns, and reproductive implications, while psychological support helps patients and partners adapt to the diagnosis and its impact on quality of life.

Our observations align with those of Chen *et al.* (2019), who analyzed 144 patients with 46,XX DSD and reported that approximately 20 % were SRY-negative (Chen *et al.*, 2019). However, very few studies have documented SRY-negative cases with detailed hormonal, imaging, and counselling outcomes from a reproductive-medicine setting. This case adds to the literature by describing a fully characterized Indian patient with SRY absence, documented microlithiasis, and multidisciplinary counselling outcomes.

We recommend karyotyping, SRY analysis, and AZF-region testing as first-line genetic investigations in non-obstructive azoospermia. Comprehensive evaluation-including hormonal profiling, high-resolution scrotal ultrasonography, and targeted molecular testing-clarifies etiology and informs individualized management.

Optimal care requires a multidisciplinary team involving reproductive medicine, endocrinology, urology, clinical genetics, and mental-health specialists. Early recognition of SRY-negative de la Chapelle syndrome ensures accurate diagnosis, prevents unnecessary procedures, supports tailored surveillance, and enables timely counselling on reproductive and endocrine options.

## CONCLUSION

SRY-negative 46,XX testicular disorder of sex development (de la Chapelle syndrome) is an uncommon but clinically relevant cause of non-obstructive azoospermia. Early cytogenetic and molecular evaluation should be performed in men presenting with small testes and azoospermia to confirm diagnosis and guide counselling. Multidisciplinary care-combining reproductive medicine, endocrinology, genetics, and psychological support-ensures accurate diagnosis, appropriate hormone monitoring, and realistic reproductive planning.
